# Diffusion Kurtosis Imaging and High-Resolution MRI Demonstrate Structural Aberrations of Caudate Putamen and Amygdala after Chronic Mild Stress

**DOI:** 10.1371/journal.pone.0095077

**Published:** 2014-04-16

**Authors:** Rafael Delgado y Palacios, Marleen Verhoye, Kim Henningsen, Ove Wiborg, Annemie Van der Linden

**Affiliations:** 1 Bio-imaging, University of Antwerp, Antwerp, Belgium; 2 Centre for Psychiatric Research, Aarhus University Hospital, Risskov, Denmark; University of Electronic Science and Technology of China, China

## Abstract

The pathophysiology of major depressive disorder (MDD) and other stress related disorders has been associated with aberrations in the hippocampus and the frontal brain areas. More recently, other brain regions, such as the caudate nucleus, the putamen and the amygdala have also been suggested to play a role in the development of mood disorders. By exposing rats to a variety of stressors over a period of eight weeks, different phenotypes, i.e. stress susceptible (anhedonic-like) and stress resilient animals, can be discriminated based on the sucrose consumption test. The anhedonic-like animals are a well validated model for MDD. Previously, we reported that in vivo diffusion kurtosis imaging (DKI) of the hippocampus shows altered diffusion properties in chronically stressed rats independent of the hedonic state and that the shape of the right hippocampus is differing among the three groups, including unchallenged controls. In this study we evaluated diffusion properties in the prefrontal cortex, caudate putamen (CPu) and amygdala of anhedonic-like and resilient phenotypes and found that mean kurtosis in the CPu was significantly different between the anhedonic-like and resilient animals. In addition, axial diffusion and radial diffusion were increased in the stressed animal groups in the CPu and the amygdala, respectively. Furthermore, we found that the CPu/brain volume ratio was increased significantly in anhedonic-like animals as compared with control animals. Concurrently, our results indicate that the effects of chronic stress on the brain are not lateralized in these regions. These findings confirm the involvement of the CPu and the amygdala in stress related disorders and MDD. Additionally, we also show that DKI is a potentially important tool to promote the objective assessment of psychiatric disorders.

## Introduction

Major depressive disorder (MDD) is a devastating disease with high prevalence and mortality. It imposes severe suffering and puts a significant burden on our society as it affects all ages and socio-economic classes. [1], [2] The core symptoms of MDD include depressed mood, cognitive deficits, anhedonia and sleep alterations. [3].

Most studies on MDD have focused on the frontal regions and the hippocampus, however, abnormalities in these regions cannot account for all symptoms of the disorder. [4] Since MDD includes a variety of distinct disease states, localized perturbations in the brain may explain the different subtypes of MDD. [5] In addition, several alternative brain regions have been found to be affected in MDD patients and animal models. Functional anomalies have been detected in subcortical regions in the brain of MDD patients [6], together with abnormalities in brain regions receiving subcortical projections. [7], [8] Furthermore, animal studies reported changes of neuron morphology such as altered arborization, spinogenesis, etc., in these brain regions and also in the amygdala, confirming the hypothesis of the involvement of multiple brain structures in the pathogenesis of depression. [9]–[11] However, there has not yet been an extensive evaluation of microstructural anatomy in the amygdala, prefrontal cortical area or the striatal area with the available state-of–the-art noninvasive neuroimaging methods.

Diffusion kurtosis imaging (DKI) is a relatively new imaging method based on the diffusion properties of water molecules and has been developed to probe the non-Gaussian diffusion characteristics. Estimation of the diffusion tensor and diffusion kurtosis tensor is based on a second order approximation of the water displacement distribution, whereas conventional diffusion tensor estimation is a linear approximation for which Gaussian distributed water displacement is assumed. The estimated diffusion kurtosis measure is dimensionless and quantifies the deviation of the water displacement profile from the Gaussian distribution and can be assumed to be a measure for microstructural complexity. [12] Since its first introduction by Jensen et al. (2005), DKI has already shown great promise in the characterization of microstructural anatomy of neuronal tissue. [13] DKI has been applied in several studies in both humans and animals, including pathological and normal conditions, such as ageing, Parkinson's disease, attention-deficit hyperactivity disorder, etc. and has been reported to be more sensitive than conventional diffusion tensor imaging (DTI) for white and gray matter alterations. [12], [14]–[17].

In a previous study, we used in vivo DKI to assess the microstructural properties of the hippocampus in the chronic mild stress (CMS) rat model. By demonstrating that mean kurtosis (MK) is the only diffusion metric that depicts the alterations of the microstructural anatomy that can be found in the hippocampus of stress exposed animals, we showed that the diffusion kurtosis metrics complements the conventional diffusion tensor metrics. In addition, we found that the shape of the right hippocampus of stress susceptible animals, which are anhedonic-like after the stress protocol, is different from stress resilient animals, which do not become anhedonic-like. Moreover, we found that the hippocampus of stress susceptible and stress resilient animals has a phenotype specific neurochemical composition, i.e. glutamate was increased in anhedonic-like animals, but not in resilient animals. [18].

The current study aimed to evaluate microstructural integrity of the aforementioned gray matter brain structures (prefrontal cortex, amygdala, caudate putamen) in subtypes of the chronic mild stress rat model for MDD, using DKI that has already demonstrated detection sensitivity in this rat model. As a control region, which has not been implicated in MDD, we included the somatosensory cortex. We designed the study to take into consideration the heterogeneity within the CMS-exposed group, i.e. the anhedonic-like and resilient group, in order to investigate the different effects of stress on brain's microstructure of stress sensitive and stress resilient animals. In addition, we examined the possibility of lateralization in agreement with previous studies reporting left/right differences in MDD and the CMS rat model. [18], [19].

## Materials and Methods

### Animals

Male Wistar rats (Taconic, Denmark), 6–7 weeks old, were singly housed with access to food and tap water ad libitum except when food and/or water deprivation was applied during the stress protocol and sucrose testing. The animals were maintained in temperature controlled rooms (21±1°C) with a 12-h light: 12-h dark cycle. After the chronic mild stress procedure the animals (n = 24) were shipped to the animal facility in Antwerp where they were housed under similar conditions. All animal procedures were performed in accordance with the European Communities Council Directive (86/609/EEC) This study was performed under the approval of the ethics committee for animal experimentation of the university of Antwerp (ID: 2008-24) and the approval of the Danish National Committee for Ethics in Animal Experimentation (ID: 2008/561-1447).

### Sucrose consumption test

The animals were first adapted to a palatable sucrose solution (1.5%). Throughout a training period of 3 weeks, the sucrose test was performed twice a week. Afterwards the test was done once a week. Over a period of two weeks a baseline sucrose consumption was established. The test comprised 1-h exposure to a bottle with sucrose solution. Prior to each test the animals were water and food deprived for 14h.

### Chronic mild stress protocol

Based on the baseline sucrose consumption, the animals were split into two matched groups and placed in separate rooms. The first group was exposed to 8 weeks of chronic mild stressors. The other group was left undisturbed. The stress procedure was adapted from [20] and is described in detail in [21]. In brief, rats were exposed subsequently to different mild stressors which each lasted between 10 and 14 hours. Mild stressors included food and water deprivation, stroboscopic light, intermittent illumination, grouping, cage tilting (45°) and soiled cages.

**Figure 1:**
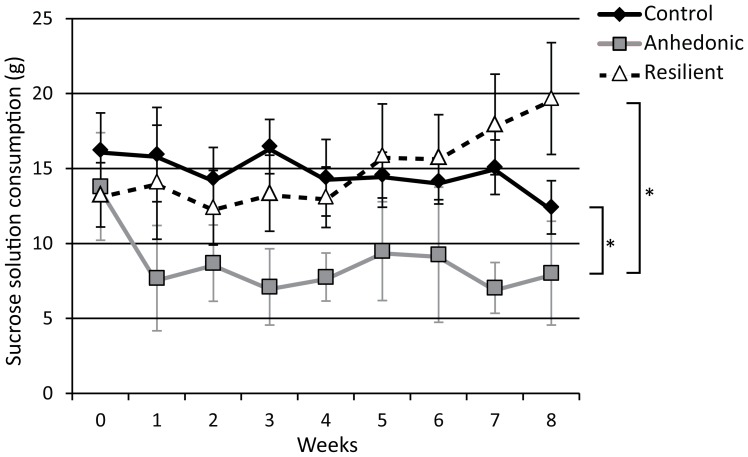
Sucrose consumption test. The rats were presented with a sucrose solution (1.5%) for one hour after 14 hours of food and water deprivation in order to evaluate their hedonic state during the CMS procedure (8 weeks). The studied animals showed a significant decrease of sucrose consumption in the anhedonic-like group, whereas the resilient animals showed no change compared with the control animals. The analysis was performed with two-way ANOVA for repeated measures. The data are presented as mean±SD. *p<.001. The figure was adapted from Delgado y Palacios *et al.* [18].

Based on the sucrose intake, the challenged group was subdivided into CMS susceptible and CMS resilient animals. From each group 8 animals were studied with MRI [22]. The studied anhedonic-like animals showed a significant decrease of sucrose consumption compared with the control animals (F_(1,14)_  = 42.792; p<.001) and with the resilient animals (F_(1,14)_  = 54.513; p<.001). The sucrose consumption of the resilient animals was not different from that of the control animals (F_(1,14)_  = .538; p = .475). (Figure 1).

### Imaging protocol

#### Experimental setup

The experiments were conducted on a horizontal bore 9.4T Bruker Biospec system (Biospec 94/20 USR, Bruker Biospin, Germany) with a 400 mT/m gradient insert. The imaging was performed using a quadrature transmit volume coil and a quadrature receive surface coil, dedicated for rats (Bruker Biospin, Germany). The system was interfaced to a linux PC running paravision 4.0 (Bruker Biospin, Germany).

For this in vivo experiment, the animals were anaesthetized with isoflurane (Isoflo, Abbot Laboratories Ltd.), administered in a gaseous mixture of 30% O_2_ and 70% N_2_ (5% for induction and 1.5–2% for maintenance). Throughout the acquisition, the animals were monitored carefully using PC-SAM (Small Animal Monitoring, SA Instruments, Inc.) to maintain constant physiological parameters during measurements. Body temperature was set at 37.5°C and was sustained using a rectal probe with feed-back to a hot air heating system (MR compatible Small Animal Heating System, SA Instruments, Inc.).

#### Diffusion kurtosis imaging

Diffusion kurtosis images were collected with a multi-slice (22 axial slices) diffusion weighted (DW) spin echo 2-shot echo planar imaging (EPI) sequence. The acquisition parameters were: echo time (TE): 25 ms, repetition time (TR): 3000, diffusion gradient pulse duration (δ): 5 ms, diffusion gradient separation (Δ): 12 ms, field of view (FOV): (35×17.5) mm^2^, acquisition matrix: 128×64, yielding an in plane resolution of (0.273×0.273) mm^2^, slice thickness: 1 mm. The DKI protocol included 7 B_0_ images and DW-images with 7 different b-values ranging from 400 to 2800 s/mm^2^, each applied along 30 directions. These images were acquired 4 times, giving a total scan time of approximately 90 minutes per subject.

#### High resolution 3D acquisition

A 3D anatomical MRI dataset of the entire brain was obtained with a T_2_-weighted 3D spin echo sequence using rapid acquisition with relaxation enhancement (RARE). The basic measurement parameters used for the RARE sequence were: TE: 14.5 ms (43.5 ms effective TE), TR: 4000 ms, spectral width: 50 kHz, flip angle: 90°, averages: 4, RARE factor: 4, FOV: (3.50×3.50×1.75) mm^3^, coronal orientation, image matrix: 256×128×64 which was zerofilled to 256×256×128, yielding a resolution of (0.137×0.137×0.137) mm^3^.

### Data analysis

#### DKI data analysis

All B_0_ and DW images were realigned to the first B_0_ image using SPM5. The estimation of the diffusion tensor (DT) and the diffusion kurtosis tensor (KT) was done by fitting the diffusion weighted signal to the DKI model as first described by Jensen et al. [12] using in-house developed Matlab (Mathworks, Natick, MA, USA) scripts [18], [23]: 
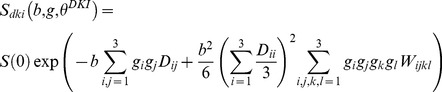
where S(0) is the signal intensity without diffusion weighting, D_ii_ is the ii^th^ element of the rank 2 DT and W_ii_ is the ii^th^ element of the rank 4 KT. From these parameters the (apparent) kurtosis in a direction g (with g =  unit length) can be computed by:
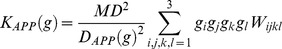



with 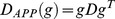



and
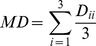



Subsequently, the axial kurtosis (AK) is the kurtosis in the main diffusion direction, the mean kurtosis (MK) is given by:
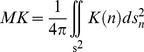



where s^2^ is the unit sphere, with n = [n1 n2 n3] ∈ s^2^, and the radial kurtosis is the mean of the kurtosis in the directions orthogonal to the direction of the main diffusion and is given by:
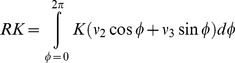



with ν_i_ the i^th^ eigenvector of the DT. In addition, the kurtosis anisotropy (KA) was calculated as: 
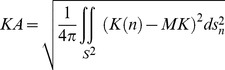



Concurrently, from the estimated DT, mean (MD), axial (AD) and radial diffusivity (RD) and fractional anisotropy (FA) parametric maps were computed [23], [24].

**Figure 2:**
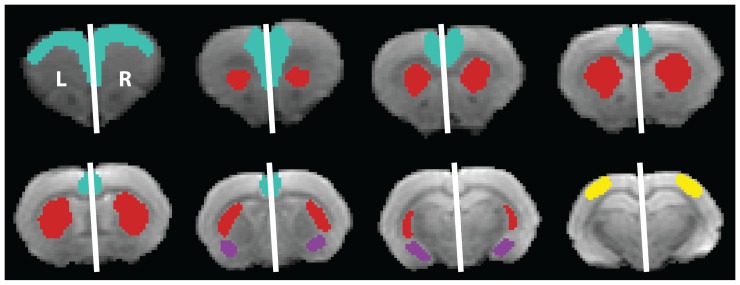
Regions of interest. The prefrontal cortex (blue), caudate putamen (red), amygdala (purple) and the somatosensory cortex (yellow) of the left (L) and the right (R) hemisphere were delineated on the B_0_ images and the estimated diffusion parametric maps, referencing to a rat brain atlas [25].

For each subject, left and right regions of interests (ROI), i.e. caudate putamen (CPu), amygdala, somatosensory cortex and prefrontal cortex were manually delineated on multiple slices (Figure 2) using the different parametric maps to minimize partial volume effects, and referencing to a rat brain atlas [25] with AMIRA software (Amira, Template Graphics Inc.). Subsequently, their corresponding mean diffusion parameters were extracted (MD, AD, RD, FA, MK, AK, RK, KA).

#### Volume data analysis

For volume analysis, the left and right CPu were manually segmented for each subject from the 3D datasets using AMIRA software (Amira, Template Graphics Inc.). The outlining of the CPu was performed by an experienced blinded researcher and reassessed by a second blinded researcher to prevent bias. The volume of the CPu was quantified based on the voxel size and the number of voxels comprising the CPu.

The volume of the prefrontal cortex and the amygdala were not examined, as these brain regions are less well-defined. The lack of sufficient anatomical landmarks to identify the prefrontal cortex and to indicate the border of the amygdala and the piriform cortex makes it difficult to accurately assess the volume of these regions.

### Statistical analysis

Statistical evaluation of the DKI data was performed using SPSS 16 (SPSS Inc.) For each parameter a two-way repeated measures analysis of variance (ANOVA) was performed per region to identify a potential lateralization effect, a main group effect or an interaction effect.

Secondly, bilateral regions that showed a main group effect, but no interaction and no lateralization effect, were analyzed as a single region. Consequently, diffusion metrics of these bilateral regions were averaged.

Finally, each parameter which showed a main group effect was compared among the three groups with one-way ANOVA and least significant difference (LSD) post hoc tests. The data was carefully checked for outliers (e.g. as a result of motion artifacts).

## Results

### Diffusion properties of the examined ROIs

**Figure 3:**
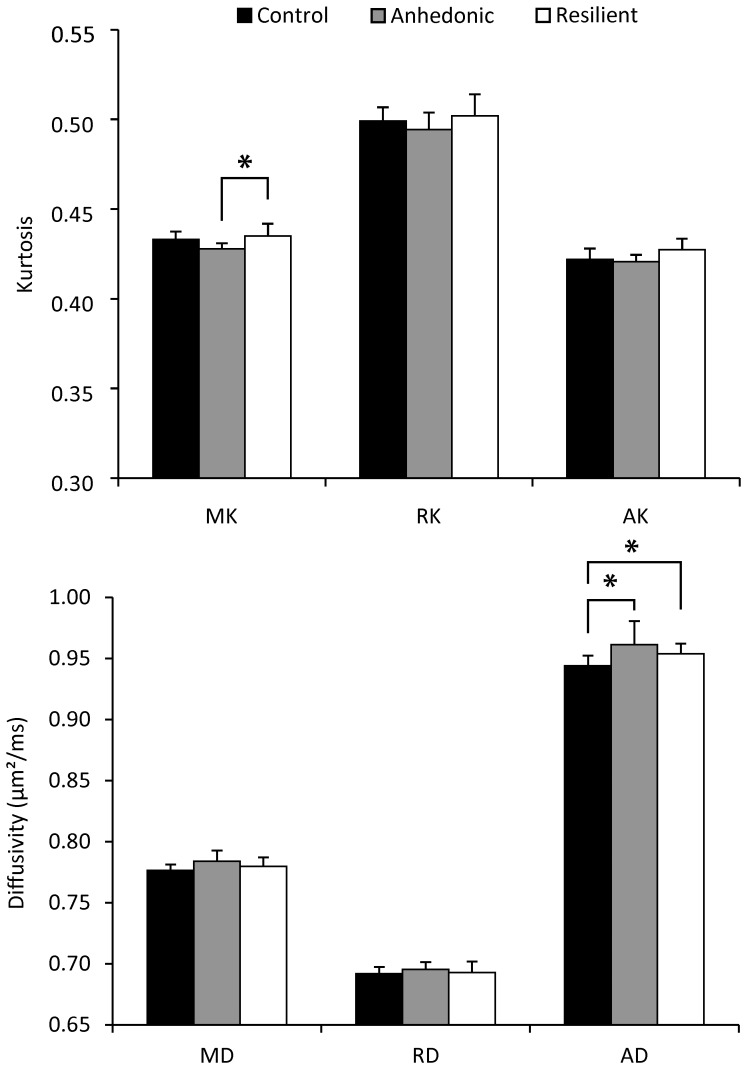
Results of the diffusion analysis of the caudate putamen. Mean (±SD) diffusion kurtosis (top) and diffusivity (bottom) parameters of the caudate putamen. One-way analysis of variance followed by post hoc least significant difference tests showed a significant increase of AD in both the anhedonic-like and resilient animals as compared with the unchallenged group. In addition, MK is significantly decreased in anhedonic-like animals as compared with resilient animals. *p<.05 AD, Axial diffusion; AK, axial kurtosis; MD, mean diffusion; MK, mean kurtosis; RD, radial diffusion; RK, radial kurtosis.

**Figure 4:**
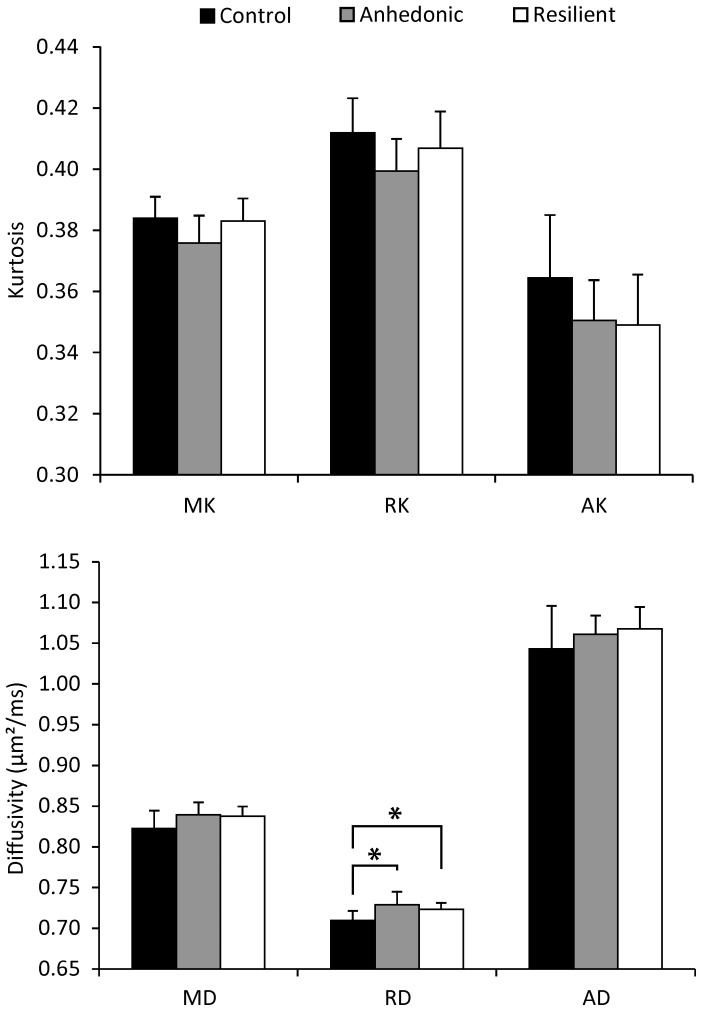
Results of the diffusion analysis of the amygdala. Groups mean (± SD) diffusion kurtosis (top) and diffusivity (bottom) parameters of the amygdala. One-way analysis of variance followed by post hoc least significant difference tests showed a significant increase of RD in both the anhedonic-like and resilient animals as compared with the unchallenged group. *p<.05 AD, Axial diffusion; AK, axial kurtosis; MD, mean diffusion; MK, mean kurtosis; RD, radial diffusion; RK, radial kurtosis.

Analysis of the DKI parameters showed that the caudate putamen (CPu) (Figure 3) and the amygdala (Figure 4) have different diffusion properties after CMS exposure. Mean kurtosis (MK) and axial diffusion (AD) in the CPu showed a significant group effect. In the amygdala, on the other hand, the radial diffusion (RD) showed a significant group effect. No significant interaction between the groups and the left/right ROI position were identified for these parameters. The other diffusion measures of the CPu and amygdala and the diffusion measures of the PFC and the somatosensory cortex did not show any significant group effect, nor did they show a significant interaction between the groups and the left/right ROI position.

The axial diffusion (AD) in the CPu was significantly increased in both CMS-exposed groups as compared with the control group. (F(2,20) = 3.846; p = .039; post-hoc tests: Control-anhedonic-like: p = .022; Control-resilient: p = .033) Furthermore, the amygdala showed an increased radial diffusion (RD) in the CMS-exposed animals as compared with the control animals. (F(2,20) = 4.396; p = .026; post-hoc tests: Control-anhedonic-like: p = .010; Control-resilient: p = .047) Anhedonic-like and resilient animals demonstrated a different mean kurtosis (MK) of the CPu. (F(2,20) = 4.263; p = .029; post-hoc test: anhedonic-like-resilient: p = .009) More specifically, the MK was decreased in the anhedonic-like animals as compared with the stress resilient animals.

### Volume analysis

**Figure 5:**
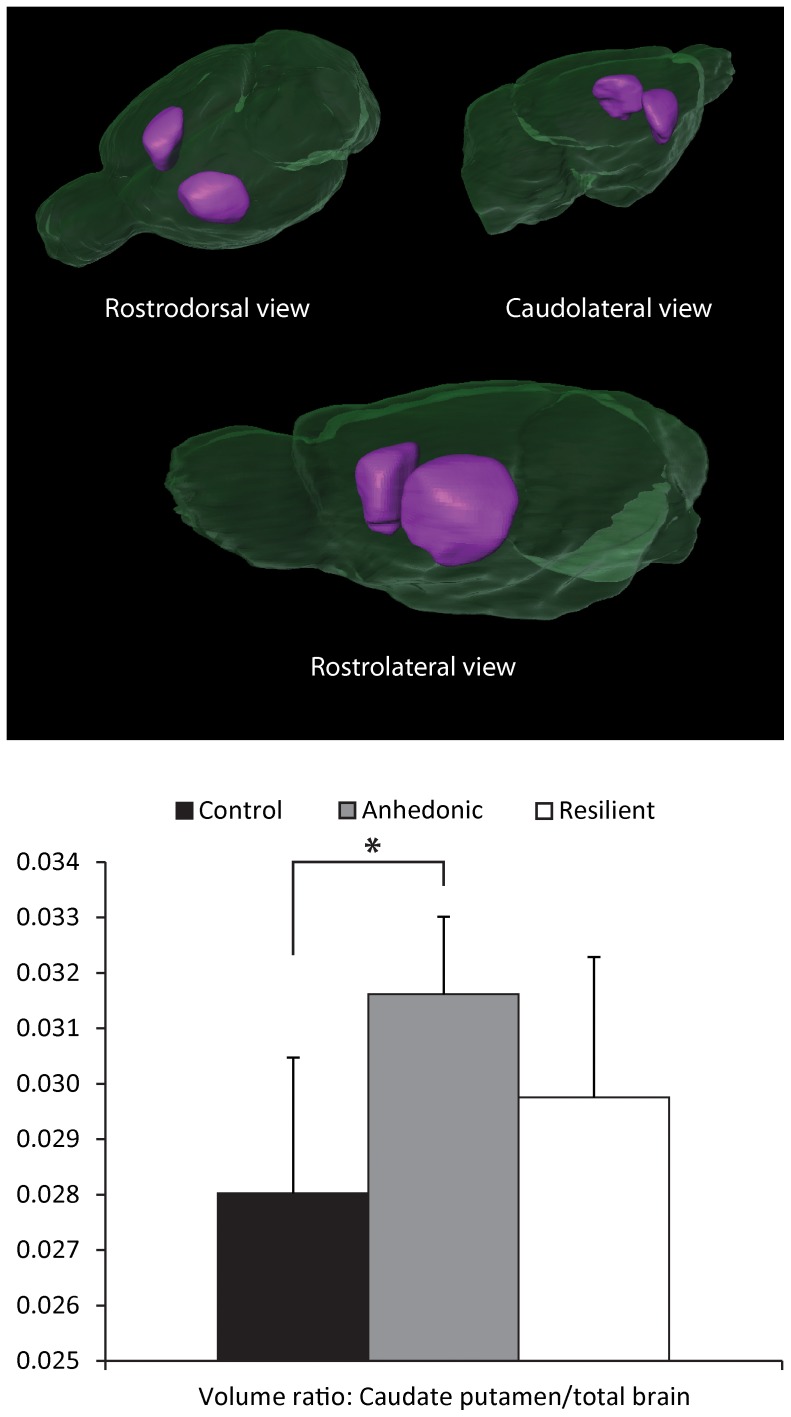
Results of the volume analysis of the caudate putamen. Top: Surface rendered MR images of the brain (green) and the caudate putamen (purple). Bottom: The caudate putamen/total brain ratio is enlarged in anhedonic-like animals as compared with control animals. *p<.05.

Main volumetric results (Figure 5) showed no significant differences in brain volume among the three groups (control: 2250±80 mm^3^; anhedonic-like: 2250±90 mm^3^; resilient: 2210±130 mm^3^; F(2,21) = .489; p = .620). No difference was found between left and right CPu. Among the three groups, the volume of the total CPu did not differ (control: 63±6 mm^3^; anhedonic-like: 71±4 mm^3^; resilient: 66±8 mm^3^; F(2,21) = 2.977; p = .073), nor was there an interaction between the left/right position of the ROI and the groups. However, anhedonic-like animals showed a significant increased CPu/brain ratio as compared with the control animals (F(2,21) = 3.640; p = .044; post-hoc tests: Control-anhedonic-like: p = .015).

## Discussion

To our knowledge, the present study shows for the first time a difference in the microstructural organization of the caudate putamen (CPu) between anhedonic-like and resilient subtypes in an animal model of depression. The principal findings of this study were that AD was increased in the CPu of the CMS exposed group as compared with the control group, and that within the CMS exposed group MK was decreased in the anhedonic-like animals as compared with the resilient animals. Stress related changes of the cytoarchitecture of the CPu are in accordance with an earlier report of dorsomedial and dorsolateral striatal reorganization after chronic stress exposure. [11] The difference in microstructure of the CPu between anhedonic-like animals and animals which seem resilient to CMS, however, is somewhat unexpected and can be a potential lead to identify alternative mechanisms that play a role in the resilience to chronic stress or stress related disorders such as MDD. On the other hand, although several studies report changes of neuron morphology and electrophysiological alterations in the prefrontal cortex area of chronic stressed rats, [26]–[28] our data did not establish any difference in diffusion properties in this region. One possibility is that the changes that have been previously reported in CMS exposed animals are not reflected in any of the diffusion parameters and therefore could not be identified. A second possibility may be the lack of anatomical landmarks on the EPI images that precludes an accurate delineation of the prefrontal cortex, which results in large variation between the subjects. Another limitation may be the incoherency of the different cortices that were comprised in the region of interest, which may have canceled a significant difference between the groups in one of the subregions.

Stress is one of the best characterized risk factors for mood disorders, including depression. [29] However, not all individuals are equally susceptible to the adverse effects of stress. Genetic make-up, environmental conditions and early life experiences constitute the vulnerability to stress. [30], [31] This has been confirmed in the CMS rat model. The CMS exposed rats segregate in a stress susceptible, anhedonic-like, group and a stress resilient group, based on behavioral analysis. [22] These two groups can also be discriminated based on neurochemical and morphometric properties of the hippocampus. [18], [32], [33] However, in a previous study, we showed that the MK of the hippocampus was similarly affected by the CMS protocol in both groups. Immunohistochemical staining with microtubule-associated protein 2 (MAP2) supported these findings and indicated that the observed kurtosis changes in the hippocampus of CMS exposed rats are associated with decreased density of somata and dendrites in the pyramidal cell layer and molecular cell layer of the hippocampus. [18] Here, we report that RD and AD of the amygdala and the CPu, respectively, also show a similar difference in both CMS exposed groups as compared with the control group.

The amygdala, a part of the limbic circuit, is implicated in mood disorders and has been reported to undergo neuronal morphology changes as a result of acute and chronic stress. [34] Spiny neurons in the basolateral amygdala complex (BLA) exhibited a significant increase of the number of spines after chronic immobilization stress (CIS). [35] Spinogenesis, however, is differently affected by CIS in the medial amygdala (MeA), eliciting a reduced spine density in medium spiny stellate neurons. [36] In addition, CIS triggers dendritical arborization in the bed nucleus of the stria terminalis and the basolateral complex of the amygdala (BLA). [9] In contrast, chronic unpredictable stress reduced the dendritical length of affected BLA neurons. [10] Since our data, which comprises the central amygdaloid nucleus and parts of the MeA and BLA, shows an increased RD, it suggests that water molecules have a more isotropic diffusion. In white matter, an increased RD has been associated with dysmyelination. [37] In other structures, this can be interpreted two ways. Dendritic atrophy may result in an increase of RD. In this case, loss of neurites results in less directional diffusion of water molecules, as the favored diffusion direction along the dendrites in a specific voxel is lost. On the other hand, the increased RD can also result from augmented arborization which drives diffusion in different directions along the dendrites. This scenario would implicate that the arborization occurs incoherently. Although further studies are needed to shed more light on the underlying mechanisms of these diffusion changes in the amygdala of CMS exposed rats, our data suggests that 1) the amygdala is affected by the applied CMS procedure, irrespective of the hedonic state, and 2) DKI of the amygdala may consolidate the diagnosis of stress related disorders.

The increase of AD in the CPu of the CMS exposed animal groups demonstrates that CMS induces microstructural reorganization of the CPu. Furthermore, MK is decreased in the CPu of the anhedonic-like animals as compared with the resilient group, suggesting that CMS has a different effect on the microstructure of the CPu in the two phenotypes of the CMS rat model. In addition, the anhedonic-like animals, but not the resilient animals, show an enlarged CPu/brain volume ratio as compared with the control group. In contrast, an earlier study in patients with late-life depression reported a decreased CPu volume. [38] The contradictory findings may therefore indicate that late-life depression and stress induced anhedonia have different pathophysiological pathways. Only limited literature is available defining the molecular processes in the striatum to discriminate resilient animals from anhedonic-like animals. One study reported that resilient animals in comparison with anhedonic-like animals show a strong neurobiological plasticity in dopamine D_2_ receptor density and mRNA expression in the striatum. [39] A second study employed a unpredictable inescapable tail shock stress (TSS) paradigm and found a decrease of thyrosine hydroxylase in the striatum of TSS exposed animals, irrespective of the behavioral consequence, whereas alpha_2_-adrenoceptor levels increased only in the striatum of stress susceptible animals. [40] Our results demonstrate that these phenotype specific molecular changes are coincided by microstructural defects. Increased AD has previously been ascribed to the loss of isotropic cells [41], however no extensive cell loss has been reported in the CPu of CMS exposed rats. On the other hand, the dorsomedial striatum (DMS) and the dorsolateral striatum (DLS) show changes of neuronal density after CMS. The DLS shows a significant increase of dendritic arborization after CMS, whereas in the DMS a trend toward arborization decrease is found. [11] Such changes of dendritic arborization have been reported to affect diffusion metrics in the cortex after prenatal cerebral ischemia. [42] Increased arborization may also account for the volume increase of the CPu. In addition, CMS induces microglial activation in the CPu, the amygdala and several other brain regions. [43] The inflammation reaction changes the morphology of the inflammatory cells and in this way may contribute to the observed diffusion and volume changes.

In contrast to the anisotropy differences reported in several DTI studies of affective disorders, we did not find FA differences in the examined regions of CMS rats. However, changes of FA in MDD patients are primarily observed in white matter structures [44], whereas the examined regions in our study have primarily gray matter properties with smaller white matter fibers passing through. The lack of structured fibers and cytoarchitectural homogeneity in these regions may result in a low sensitivity of FA to detect anisotropy changes.

The present study has also some limitations. First, because DKI demands high b-value diffusion weighted images, it is sensitive to noise. Consequently, the resolution of the acquired images is suboptimal to sustain sufficiently high signal-to-noise ratios and to minimize acquisition time. This may have led to partial volume effects, which could have reduced the sensitivity of the analysis. Second, due to the limited number of subjects, subtle differences between groups might not appear significant. Third, to examine the underlying mechanisms leading to the reported diffusion changes an extensive histological analysis is necessary.

In conclusion, our findings point out a potentially important role for the CPu in the development of anhedonia. Moreover, DKI metrics changes and stress-induced enlargement of the CPu may constitute a fingerprint for stress-induced depression in vulnerable individuals. Hence, future mood disorder studies focusing on the striatal region may reveal possible important underlying mechanisms for the development of stress related disorders.
